# Pensions to Asylum Officials

**Published:** 1904-01-16

**Authors:** 


### PENSIONS TO ASYLUM OFFICIALS.

THE county and borough asylums of England and Wales now number eighty or more, and taking them all in all they are institutions of which the nation may well be proud. In them the care of the insane has been carried to almost the highest point of perfection and of late years, at any rate, the cure of the patients has not lagged far behind the care. To maintain these good points at high-water mark should be the desire of the State, and no reasonable means should be neglected by it to ensure the continuance of that high level.

After much study of the question and after some personal acquaintance with the inner working of asylums we are satisfied that few conditions could have a greater value than that all officials who have the care of patients should have as a matter of right the certainty of a retiring allowance for their old age, or earlier than that in case of permanent ill-health. As things are at present pensions are permissive only, and this asylum service is the only one which can be in any way identified with the civil service in which the granting of pensions is permissive and not compulsory. Why this extraordinary state of things obtains in a department where the duties are more irksome to perform, more trying to the mental and bodily health than any other is strange almost to incredibility; but such is the law. The old Lunacy Act permitted the granting of pensions not exceeding two-thirds of salary and allowances after twenty years' service, and 50 years of age; and this granting was in the hands of the Committee of

Visitors. The next Act reduced the length of service to fifteen years; but it burdened the grant with the consent of quarter sessions, a consent which was, however, never refused. The last Lunacy Act left the question precisely as it had been before, but then it was generally felt by officials that when asylums were placed under a committee of the elected County Councils the hope of receiving a pension—a hope that was practically a certainty—might be jeopardised, and although this fear has been proved to be, in the vast majority of cases, a groundless one, it has not been altogether so for several asylum committees have publicly intimated that pensions will not be granted by them. It is therefore, we repeat, the duty of the State to step in and set this question at rest, and this could easily be done by inserting a clause in the next Lunacy Act Amendment Bill, which Bill is not likely to be very long delayed.

We have made inquiries at every county and borough asylum in England and Wales, and although we have not received answers to all our inquiries which related to facts only and not to opinions, we have had a sufficient number of answers to enable us to lay before our readers the prevailing rules in a majority of cases. In at least 13 asylums the committee of visitors have, within the limits of the Act, framed pension schemes of their own, and this is a plan much to be commended as it enables officials to learn exactly, or nearly, what may be in store for them. These schemes vary somewhat in detail, but the principle is the same in all; and as a rule a very proper distinction is drawn between officials who are in constant attendance on the insane and those who are not. It is with the first class that we are chiefly concerned. In one asylum the amount of pension given is from one twenty-fifth to one-fiftieth of salary and emoluments for every year of service. In another it is from one-fortieth to one-fiftieth. In another it is from one-third to two-thirds of salary and allowances, and the recipient must be 55 years of age. It would seem that the average is rather more than one-fortieth for every year of service. It cannot be said that this is excessive—indeed, in some instances, it would not be sufficient to live on.

To come somewhat more into detail, it may be stated that the lowest pension received by any medical superintendent has been £350, and the highest £1,050, the average about £700; chaplains have received from £50 to £200, the average being about £100; chief male attendants from £45 to £84, with an average of £60; chief female officers from £20 to £100, with an average of £59; ordinary male attendants from £18 to £50; and female attendants from £15 to £43. Doubtless in all these cases the Committee of Visitors took into consideration the length of service, the value of the service, and the age of the official to be pensioned; hence the great discrepancy existing in the amounts given. In public asylums it would hardly be possible to point to two institutions which are exactly alike in respect to the duties allotted to the members of the staffs, the salaries received, and the energy with which the work is done, therefore no good end could be obtained by comparing the pensions given in different asylums, and hence the desirableness of giving the Committee of Visitors latitude in granting pensions, but while fixing the maximum of pensions which can be granted, it is essential to fix the minimum also. In addition to this latitude, it would be a wise provision to allow the Committee to add in certain cases a few years to the length of service. For instance, an officer appointed, say, at the age of 35 retires at 55 after 20 years' service. If he has been a highly paid official, the pension may be sufficient; but if in a subordinate position it may be quite insufficient. In connection with this, it should be borne in mind that an official who has been appointed from the asylum of one county loses all the service therein when he takes office in another.

Whatever may be done by Parliament in the way of securing pensions to asylum officials, it is certain that compulsory retirement should go hand-in-hand with compulsory pensions. In some of those asylums which have formulated schemes, the age limit is 60, and in others it is 65. The former age should not be exceeded.

To the ratepayers the granting of pensions should commend itself, as it is incomparably more economical to them than the only other satisfactory alternative, namely, to increase salaries and wages all round.

